# Six Weeks of Confinement: Psychological Effects on a Sample of Children in Early Childhood and Primary Education

**DOI:** 10.3389/fpsyg.2020.590463

**Published:** 2020-10-08

**Authors:** Marta Giménez-Dasí, Laura Quintanilla, Beatriz Lucas-Molina, Renata Sarmento-Henrique

**Affiliations:** ^1^Research and Psychology in Education, Complutense University of Madrid, Madrid, Spain; ^2^Methodology of Behavioral Sciences, National University of Distance Education (UNED), Madrid, Spain; ^3^Developmental and Educational Psychology, University of Valencia, Valencia, Spain

**Keywords:** confinement, psychological effects, COVID-19 health crisis, early childhood, primary education, Spanish children

## Abstract

Spain has been one of the countries most affected by the health crisis derived from COVID-19. Within this country, the city of Madrid has registered the highest number of infections and deaths. This circumstance led to the adoption of strict confinement measures for a period of 6 weeks. The objective of the present study was to investigate the psychological effects that this confinement has had on the psychological well-being of a sample of children from Madrid. A total of 167 families with children aged between 3 and 11 years participated in this study. The parents evaluated the children through the System of Evaluation of Children and Adolescents (SENA) scale in the month of February and refilled part of the same scale after the children had spent between 4 and 6 weeks confined. The comparison between the two measures showed no change among the 3-year-old children. However, change was observed among the 6–10-year-old. Children in Primary Education obtained lower scores in dimensions related to self-regulation (emotional, attentional, and behavioral) and in willingness to study. The results are discussed in light of the situation experienced between the months of March and May 2020.

## Introduction

Spain has been one of the countries most affected in the world by the health crisis caused by the Severe Acute Respiratory Syndrome Coronavirus 2 (SARS-CoV-2). Within this country, Madrid has been the city where more confirmed cases of contagion and more deaths have occurred. This situation makes the city of Madrid one of the places in the world most affected by this health crisis and where the restrictions have been the harshest. The entire population of this Autonomous Community and this city (around 6 million inhabitants, INE, 2019) has suffered strict confinement measures for 2 months, which have also affected children.

On the 11th of March, all educational centers in Madrid closed, from Early Childhood Education to University levels. On the 14th of March, the state of alarm was declared and people were prohibited from leaving their homes except for essential supply services (Real Decreto 463/2020, 2020). For children, this confinement lasted until the 25th of April (Real Decreto 492/2020, 2020). After 6 weeks and 3 days at home, children were allowed to go outside for an hour a day. This outing regime was maintained until the 25th of May, date on which meetings with friends and family in private homes up to a maximum of 10 people were authorized (Real Decreto 537/2020, 2020). Thus, the most demanding isolation measures for the children of Madrid extended over a period of 6 weeks and the measures with a partial relief of 1 h of outing per day for an additional 4 weeks. In total, the children of Madrid have lived confined or with only 1 h a day of outing for a total of 10 weeks.

This novel situation in our world, due to the unexpectedness of the measure and its duration, may have had some effect on the psychological well-being of children. Previous research shows that social isolation can cause some difficulties such as depression, anxiety, emotional problems, or sleep disorders (Holt-Lunstad et al., 2015; Urbina, 2020). Some studies have found alterations related to an increase in anxiety in between 16 and 30% of the population in the face of health crises already experienced, such as SARS, Ebola, or swine flu (Rubin et al., 2010; Shultz et al., 2016; Satcher and Kenned, 2020). Other very recent studies have shown that the duration of confinement and the associated emotions can generate significant psychological stress, as well as anxiety, delusional thinking, obsessions, or rumination in vulnerable people (Brooks et al., 2020; Mucci et al., 2020).

Similarly, previous research on the effects of social isolation in children shows important effects on aspects, such as feelings of sadness, anger, frustration, and apathy (Biordi and Nicholson, 2013; Brooks et al., 2020). Other indicative aspects of well-being and regulation during childhood have also been found to be altered, such as sleep patterns, potty training, or challenging behaviors (Simon and Walker, 2018). Changes have also been observed in the levels of anxiety (increased fear, worry, obsession, or rumination) and depression (depressed mood, lack of interest and motivation, or sadness; Teo et al., 2013; Urbina, 2020). A study evaluating the psychological effects of pandemics in children and adults found that the risk of posttraumatic stress was four times higher for children who had suffered confinement measures compared to those who had not (Sprang and Silman, 2013).

There are very few published studies on the effects of confinement derived from the SARS-CoV-2. Jiao et al. (2020) report a study with families of 320 Chinese children aged between 3 and 18 years. The families evaluated the frequency of clinical symptoms through an online questionnaire. The results showed that the most frequent symptoms in the entire sample were inattention, irritability, and clinging. More specifically, the older children (6–18) showed greater inattention and persistent inquiry, while the younger ones (3–6) showed more clinging and fear that family members could contract the illness. In Europe, a study prior to ours among Italian children showed a significant impact on the psychological well-being of children (Pisano et al., 2020). Italy and Spain have been two comparable cases in confinement measures, number of infections, and deaths. Pisano et al. (2020) designed a questionnaire for parents to assess the perceived changes in children aged between 4 and 10 years during the 1st month of confinement. The sample consisted of 5989 participants. Between 20 and 30% of parents said that their children showed more symptoms, such as irritability, excessive demands, sleep problems, mood changes, or fear. Around 43% indicated that their children seemed more apathetic when performing their usual activities. Some positive effects were also found. In fact, 92% stated that their children had been able to adapt to the restrictions imposed by the pandemic, 49% indicated that their children seemed more reflective, and 31% indicated that they were calmer.

In Spain, we also found a study, prior to ours, in which a group of sociologists designed a questionnaire for children to answer online (Martínez Muñoz et al., 2020). This questionnaire was available between the 21st of March and the 7th of April for boys and girls aged between 8 and 17 years. It is important to note that the questionnaire was completed during the most difficult days of the crisis, when the number of daily deaths was close to a 1,000 people. A total of 425 boys and girls answered the questionnaire, the majority aged between 10 and 14 years. The sample was made up of children residing in different communities in Spain. Most of the sample expressed a high level of satisfaction and well-being in their daily lives. This result suggests that the sample was made up of children belonging to normalized environments and without problems of social exclusion, poverty, etc. The results of this work are interesting and show, according to previous studies, some of the psychological effects that confinement and the health crisis had at that time on the children. Thus, the most frequent feeling that the participants said they experienced every day or quite a lot was boredom (61% of the sample), followed by worry (36%), sadness (28%), and fear (16%). The main concern of the children was that a member of their family would fall ill or die (83%). A very relevant fact has to do with the relationship between subjective well-being and academic performance. There was a negative relationship between perceived well-being and the feeling of being overwhelmed by academic tasks. In this sense, 26% of children said they felt overwhelmed by the amount of academic work sent by their teachers and 60% said they felt tired from working so much. These two results show that, on the whole, 85% of children perceived the work demanded by their teachers as exaggerated or inadequate and this perception affected their sense of well-being. Finally, children expressed joy during these weeks for being able to spend more time with the family (45%), having more time to play (23%), having free time (8%), and not having to get up early and not go to school (4%). These results clearly show that Spanish children overwhelmingly demand more time for their family life, for themselves, and for resting.

These previous results lead us to think that Spanish children were able to experience negative changes and, perhaps, also positive changes derived from the absence of school, the greater availability of time, and family life. However, one of the main problems of the studies that began to evaluate once the crisis had started is the absence of a previous measure with which to compare the changes experienced.

The objective of the present study is to evaluate the changes that the confinement situation experienced between the 11th of March and the 25th of April could have caused on a sample of children residing in the Community of Madrid. Our study is the result of a particular circumstance that has allowed us to compare the changes between a previous measure of adaptation and psychological well-being, collected during the month of February, and a subsequent measure taken during the most restrictive period of confinement. In this sense, we believe that we can provide a reliable assessment of the effects that the health crisis and confinement has had on children in Madrid after 6 weeks of restrictive measures.

## Materials and Methods

### Participants

A total of 167 families with children aged between 3.2 and 11.1 years (42% girls, mean age of 7 years and 2 months, SD = 2.64, and range = 3.2–11.1) participated in the study. The sample was divided into two age groups: preschool families (*M* = 3.9, *SD* = 0.6, range = 3.2–6.2) and primary families (*M* = 8.6; *SD* = 1.7, range = 6–11.1). From this sample, 113 families completed a questionnaire before and during confinement (75 from Primary Education). The rest of the participants (54) only completed the questionnaire during confinement (42 from Primary Education). The convenience sample was obtained thanks to the fact that the schools were participating in a research project related to the promotion of emotional skills when the pandemic struck. Children went to two public schools of the northern area of Madrid (Spain) and resided in middle and upper middle class neighborhoods.

### Instrument

The questionnaire used was the System of Evaluation of Children and Adolescents (SENA, Fernández-Pinto et al., 2015), validated and scaled for the Spanish population. This instrument offers a comprehensive assessment of emotional and behavioral problems for ages between 3 and 18 years. As it is a very broad questionnaire, with more than 100 items, answering it fully during the confinement involved a too demanding task for parents with school-age children. For this reason, those dimensions of the questionnaire that evaluated aspects related to psychological adjustment were selected. The selected scales were Attentional Problems, Depression, Challenging Behaviors, Emotional Regulation, Hyperactivity, and Willingness to study (the latter only for the Primary Education version). The score for each item ranged from 1 (never or almost never) to 5 (always or almost always). For all dimensions, except for Willingness to study, the lowest scores indicate absence of problems and scores above 3 indicate the presence of some type of difficulty. For the Willingness to study scale, a score lower than 3 indicates a problem.

To the selected scales of the questionnaire, we added an open-ended question, so that the families could comment on any aspect that had not been included in the questionnaire. The instruction was “Comment here on anything you have observed, any change you have noticed in your child since the confinement began that seems significant to you and has not been included in the previous questions (for example, eats too much, has nightmares, complains of headaches, is very afraid, etc.).”

### Procedure

As a consequence of the confinement caused by COVID-19 and the closure of the educational centers in Madrid decreed on the 11th of March 2020, the research project in which we had been working with the two educational centers in the northwestern area of Madrid had to be discontinued. However, according to the schedule of our research project, before the closing of the educational centers we had carried out assessments on different aspects of the participating children. The families had completed the assessment tests related to the children during the month of February. Based on these previous assessments, a month and a half later and after having spent between 4 and 6 weeks of confinement, we again asked the families to fill out a part of the questionnaire. All the questionnaires were answered by the parents between the 8th and the 25th of April 2020, granting their consent to participate in the study. Both the pretest and posttest questionnaires were answered online. This research was approved by the Deonthological Committee of the Faculty of Psychology of the Complutense University of Madrid. The principals and the families of the participating schools were informed of the purpose of the study by the research team. Parents signed the appropriate consent forms. Both the university and the schools followed the protocols for applying the ethical procedures that regulate research in Spain.

### Data Analyses

As we mentioned before, the families had answered the questionnaire during the month of February, a few weeks before confinement. This situation could sensitize parents when answering the questionnaire in the second round. On the other hand, some families did not answer the pretest (T1) but did answer the questionnaire during confinement (posttest/T2). To control the possible sensitivity to the test of the participants with pretest, the scores of both groups were compared – with and without prior assessment in the T2 measure. To do so, we used a MANOVA in which the main factor was whether or not the assessment was carried out before confinement. With this analysis, we wanted to rule out that the T1 measure had interfered with the T2 measure in that group.

To assess whether there were differences in the psychological adjustment between before and during confinement, we carried out a repeated-measures (RM) ANOVA in which the psychological adjustment was contrasted in the two measures, observing the possible differences in age and sex. Given that the SENA questionnaire has different items for children in Early Childhood Education (3–6 years) and Primary Education (6–12 years), we carried out the statistical analysis for each group separately. We used SPSS 24 for these analyses.

The open-ended question was coded into broad categories of change, that is, if the change reported by the family indicated a worsening of the child’s condition, an improvement, or an absence of change. Likewise, the symptoms described by the families were grouped into several categories. Subsequently, the percentage of responses was calculated for both the Early Childhood and Primary Education groups. The objective of this open-ended question was to obtain a more accurate description of the families’ perception of their children’s changes during confinement.

## Results

The preliminary analysis carried out to verify whether previously applying the questionnaire had sensitized the participants indicated that the difference between the groups with and without prior assessment was not significant, either for the Primary Education group [*F*(6, 110) = 1.37, *p* = 0.231, $\eta_{p}^{2}=0.07$] or for Early Childhood Education [*F*(5, 44) = 0.517, *p* = 0.762, $\eta_{p}^{2}=0.05$]. This result allowed us to consider with greater guarantee that the possible differences between the pretest and the posttest assessment were not biased.

Comparison between the pretest and posttest scores for the Early Childhood Education group indicated very little variation in the mean scores of the five dimensions (see Table 1). The ANOVA (RM) analysis effectively indicated that the differences were not significant [*F*(5, 44) = 1.6, *p* = 0.19, $\eta_{p}^{2}=0.19$]. There were also no gender [*F*(5,30) = 0.620, *p* = 0.686, $\eta_{p}^{2}=0.09$] or age [*F*(5,30) = 0.43, *p* = 0.82, $\eta_{p}^{2}=0.39$] differences.

**Table 1:** Scores obtained before and during confinement in the dimensions evaluated in children in Early Childhood and Primary Education.

	Early childhood education (*N* = 38)				Primary school (*N* = 75)			
	BC		DC		BC		DC	
	M	SD	M	SD	M	SD	M	SD
Attention problems	2.11	0.68	2.31	0.76	2.21	0.81	2.42	0.83
Depression	1.19	0.24	1.28	0.45	1.40	0.45	1.60	0.64
Hyperactivity and impulsivity	2.64	0.69	2.67	0.83	2.30	0.77	2.66	0.89
Emotion regulation	2.26	0.67	2.43	0.92	2.15	0.74	2.42	0.93
Challenging behavior	2.41	0.77	2.44	0.87	0.71	0.75	0.75	0.75
Willingness to study					3.02	0.65	2.38	0.74

BC, before confinement; DC, during confinement.

On the contrary, for the Primary Education group of children, some differences were observed between the mean scores obtained before and during confinement (see Table 1). In general terms, the scores suggest that there was normality regarding the dimensions evaluated. In the pretest evaluation, the scores were mostly rather low, while some mean scores increased during confinement and Willingness to study decreased.

The statistical analysis indicated that there were no significant differences due to gender [*F*(6, 55) = 1.49, *p* = 0.23, $\eta_{p}^{2}=0.13$] or age [*F*(6, 55) = 1.33, *p* = 0.09, $\eta_{p}^{2}=0.13$], yet significant differences were found due to confinement [*F*(6, 55) = 5.89, *p* < 0.001, $\eta_{p}^{2}=0.39$] with a significant effect size.

The contrasts for each dimension indicated significant differences in the Attention [*F*(1, 60) = 5.74, *p* = 0.02, $\eta_{p}^{2}=0.08$], Willingness to study [*F*(1, 60) = 24.74, *p* < 0.001, $\eta_{p}^{2}=0.08$], Emotional Regulation Problems [*F*(1, 60) = 6.35, *p* = 0.01, $\eta_{p}^{2}=0.09$], and Hyperactivity and Impulsivity [*F*(1, 60) = 13.62, *p* < 0.001, $\eta_{p}^{2}=0.18$] scales. In the other two scales, there were no significant differences. Thus, the scores in the Depression scale [*F*(1, 60) = 2.5, *p* < 0.11, $\eta_{p}^{2}=0.04$] and Challenging behavior [*F*(1, 60) = 0.37, *p* < 0.54, $\eta_{p}^{2}=0.04$] scales did not vary.

**Figure 1:**
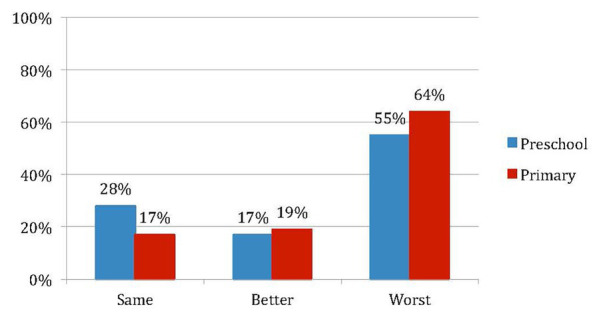
Percentage of changes reported by parents during confinement (Preschool *N* = 29; Primary *N* = 58).

The qualitative analysis of the responses was carried out on the voluntary comments of the parents. It should be noted that only 62% of the participants answered this open-ended question. The comments were classified into three categories: those that indicated an improvement in the child’s general condition, those that indicated a worsening, and those that did not show any significant change. Figure 1 shows the percentage of responses for each category in each age group.

Families reported a higher percentage of children whose psychological state worsened, both in Early Childhood (55%) and in Primary Education (64%). However, this result was higher in Primary Education, where only 36% of families indicated that children did not change (17%) or had improved (19%). When comparing Early Childhood and Primary Education, it is observed that in Early Childhood, the percentage of children who did not experience changes was higher (28%) and the percentage of children who improved their psychological state (19%) was similar. Taken together, these results are consistent with those obtained through the questionnaire, showing, on the one hand, that although children were affected by the situation of confinement, those in Primary Education suffered this situation more than those in Early Childhood Education, and on the other, that a significant percentage of children in both cycles improved their psychological state. In addition to these percentages, the behavior or type of symptomatology described by families in the open-ended question was also classified. Table 2 lists the percentages of the types of behaviors that parents perceived in their children.

**Table 2:** Percentage (frequencies in brackets) of behavioral changes or symptoms observed by families during confinement.

	Early childhood education *N* = 16	Primary school *N* = 65
Emotional regulation	38 (6)	46 (30)
Sleep, eating habits	19 (3)	20 (13)
Sphincter control/potty training	25 (4)	-
Attentional and school difficulties	6 (1)	18 (12)
Somatizations	6 (1)	8 (5)
Hyperactivity	6 (1)	5 (3)
Misses being in school	-	3 (2)

In the Early Childhood group, families reported overall greater difficulties in emotional regulation (he/she is more irritable, has more mood swings, etc.), in sleeping and eating patterns (he/she does not want to sleep alone, has trouble falling asleep, has nightmares, eats more, etc.) and in potty training (he/she wets the bed again at night, has had a potty accident during the day, etc.). In the Primary Education group, families also mentioned these three types of behaviors or symptoms, but they also mentioned attentional difficulties and, above all, school difficulties. Finally, some families indicated positive changes, referring to improvements in mood (he/she is happy, is calmer, etc.) and to the positive effect of the greater availability of free time (he/she has more time to play, has more time for him/herself, etc.) and family time (he/she is delighted to be with us, enjoys playing with his/her sibling very much, has strengthened the bond with his/her siblings, etc.).

## Discussion

The objective of the present study was to verify whether the situation of 6 weeks of strict confinement experienced in Madrid as a consequence of the SARS-CoV-2 health crisis had caused a change in the psychological well-being of children. The results show significant changes in most of the indicators evaluated in the older children of the sample (6–11 year-old). These changes, however, were not observed in the younger children of the sample (3-year-old). More specifically, children aged between 6 and 11 scored higher in emotional regulation difficulties, attentional difficulties, hyperactivity, and impulsivity. Willingness to study was the scale on which the worst result was obtained. In this dimension, the greatest difference was observed between the pretest and posttest scores. On the contrary, no changes were found in the Depression or Challenging behaviors scales.

In general, the results obtained are congruent with those found in previous studies on the effects of social isolation on children and also with the few studies that have been carried out on the particular situation of confinement during SARS-CoV-2 health crisis (Jiao et al., 2020; Pisano et al., 2020). Despite this coincidence, it is necessary to highlight that the two previous studies mentioned found a significant increase in symptoms in children aged from 3 to 4 years, respectively; in our case, this difference was delayed until the age of 6 years. It is possible that the sample size may have influenced these differences.

One of the most striking results of the present study is the greater difficulties that Primary school children seem to experience when carrying out school tasks. This result coincides with the school difficulties expressed by the children in study of Martínez Muñoz et al. (2020). Despite the fact that the mean age of the sample in this study was 12 years, it should be highlighted that 85% of children expressed difficulties in adequately performing school tasks as well as excessive demand on behalf of teachers. Our results also show that the dimension in which Primary children obtained worst results was related to academic performance. In this sense, it is necessary to take into account the difficulties that online teaching may pose for Primary children. As Rogero-García (2020) points out, distance learning can become a fiction in which no one can fulfill their role, but in which frustration and stress of teachers, students, and families will grow.

The difference observed in the present study between Early Childhood and Primary Education children shows that, in some way, young children are more protected from reality than older children. This protection comes, on the one hand, from the family and, on the other, from the cognitive system of young children. In this sense, the care that young children receive from their families should facilitate the continuity of their lives, turning confinement into a time similar to that of holiday time spent at home or periods of illness that are so frequent in young children. Furthermore, the toddler’s cognitive system operates largely within the framework of fiction (Harris, 2000). Fiction is a fundamental element for development and learning that in this age group has a very relevant role. It is possible that the time and cognitive effort that children dedicate to fiction constitutes a protective element of psychological well-being in this circumstance. It is also possible that these two protective factors have been less present in the lives of children aged 6, 8, and 10 years during confinement and that part of their psychological deterioration may be due to the progressive decrease in pretend play that is experienced from the age of 7, the more conscious perception of reality, and the lesser need for constant attention from the adults.

Despite the fact that Early Childhood children did not show a significant worsening, when the families described the observed changes, they mentioned similar symptomatology to that manifested by Primary Education children. These changes have to do mainly with regulatory skills and are manifested in behaviors related to Executive Functions (i.e., emotional regulation, attentional control, hyperactivity, and impulsivity). Likewise, despite the fact that families pointed out regulation problems, around 40% of families observed that children were not affected or had even improved during the situation of confinement. This improvement situation, in which around 20% of the children are, together with the comments of the families that noted that children were happy to be able to have more time and enjoy family life constitutes a call of attention on the impact that school days and daily activities can have on the well-being of children. Although the children who were not affected by or even appreciated the situation of confinement were not a majority, they constitute a significant percentage and it would be desirable to make the appropriate adjustments to optimize their development.

Finally, keeping in mind the reality we will probably have to face this following academic year, with the difficulties that online teaching can pose for children of these ages, the decrease in the willingness to study that has been observed in these 6 weeks of confinement, and the stress that the academic burden can pose for children, it would be necessary for the responsible authorities in education to design an academic course focused on the essential contents, adapting the demands to the real working and learning abilities of the children, in which the workload is reduced, the stress is decreased, and the psychological well-being is promoted. The impact that this health crisis may have on the psychological well-being of future generations is still unknown, but the first studies carried out in Spain coincidentally indicate an increase in academic difficulties. The design of the next course can be a very relevant variable. Education managers, school directors, and teachers have an important role to play in the coming months (Jansen et al., 2020).

## Limitations and Future Lines of Research

This study has some limitations that need to be considered. Firstly, the sample size is small and it is a convenience sample. In the future, it will be necessary to design studies with representative populations and larger samples in order to generalize the results. Likewise, it would be necessary to carry out studies with vulnerable populations. Secondly, the instrument applied is relatively small. It would have been desirable to be able to obtain broader assessments that consider other dimensions. Thirdly, it would also be necessary to carry out medium‐ and long-term follow-up studies to understand the scope and maintenance of the changes observed in these 1st week of confinement. Moreover, the long-term impact on mental health together with the need to monitor children and adolescents’ well-being are important issues to consider for future research (Lee, 2020).

## Data Availability Statement

The raw data supporting the conclusions of this article will be made available by the authors, without undue reservation.

## Ethics Statement

The studies involving human participants were reviewed and approved by Comisión Deontológica, Facultad de Psicología, UCM. Written informed consent to participate in this study was provided by the participants’ legal guardian/next of kin.

## Author Contributions

MG-D designed the study. MG-D and LQ have collected the data. LQ has analyzed data. MG-D and LQ have written the first draft. BL-M and RS-H have reviewed and completed the manuscript. MG-D did the submission. All authors contributed to the article and approved the submitted version.

### Conflict of Interest

The authors declare that the research was conducted in the absence of any commercial or financial relationships that could be construed as a potential conflict of interest.
